# Higher Incidence of BK Virus Nephropathy in Pediatric Kidney Allograft Recipients with Alport Syndrome

**DOI:** 10.3390/jcm8040491

**Published:** 2019-04-11

**Authors:** Young Hoon Cho, Hye Sun Hyun, Eujin Park, Kyung Chul Moon, Sang-Il Min, Jongwon Ha, Il-Soo Ha, Hae Il Cheong, Yo Han Ahn, Hee Gyung Kang

**Affiliations:** 1 Department of Pediatrics, Seoul National University College of Medicine and Seoul National University Hospital, Seoul 03080, Korea; tccyh@hanmail.net (Y.H.C.); inthy@naver.com (H.S.H.); eujinpark@hallym.or.kr (E.P.); ilsooha@snu.ac.kr (I.-S.H.); cheonghi@snu.ac.kr (H.I.C.); 2 Department of Pediatrics, St. Vincent’s Hospital, College of Medicine, The Catholic University College of Korea, Seoul 06591, Korea; 3 Department of Pediatrics, Kangnam Sacred Heart Hospital, Hallym University College of Medicine, Seoul 07441, Korea; 4 Department of Pathology, Seoul National University College of Medicine, Seoul 03080, Korea; blue7270@gmail.com; 5 Department of Surgery, Seoul National University College of Medicine, Seoul 03080, Korea; surgenmsi@gmail.com (S.-I.M.); jwhamd@snu.ac.kr (J.H.); 6 Transplantation Research Institute, Seoul National University College of Medicine, Seoul 03080, Korea; 7 Department of Pediatrics, Seoul National University Bundang Hospital, Seongnam 13620, Korea

**Keywords:** BK virus, BK virus nephropathy, kidney allograft, transplantation, Alport syndrome, children

## Abstract

A retrospective review was performed to assess the risk factors and outcomes of BK virus infection and nephropathy (BKVN), an early complication in pediatric kidney allograft recipients. The study investigated the incidence, risk factors, and clinical outcomes of BK viremia and BKVN in a Korean population of pediatric patients who received renal transplantation from 2001–2015 at the Seoul National University Hospital. BKVN was defined as biopsy-proven BKVN or plasma BK viral loads >10,000 copies/mL for >3 weeks. BK viremia was defined as a BK viral load >100 copies/mL in blood. Among 168 patients assessed for BK virus status, 30 patients (17.9%) tested positive for BK viremia at a median of 12.6 months after transplantation. BKVN was diagnosed in six patients (3.6%) at a median of 13.4 months after transplantation. Three of the six BKVN patients had Alport syndrome (*p* = 0.003), despite this disease comprising only 6% of the study population. Every patient with BK viremia and Alport syndrome developed BKVN, while only 11.1% of patients with BK viremia progressed to BKVN in the absence of Alport syndrome. Multivariate analysis revealed that Alport syndrome was associated with BKVN development (hazard ratio 13.2, *p* = 0.002). BKVN treatment included the reduction of immunosuppression, leflunomide, and intravenous immunoglobulin. No allografts were lost in the two years following the diagnosis of BKVN. In summary, the incidence of BKVN in pediatric kidney allograft recipients was similar to findings in previous reports, but was higher in patients with underlying Alport syndrome.

## 1. Introduction

BK virus (BKV) is a polyomavirus that resides in the urogenital tract as a latent infection [1]. The seroprevalence of BKV in the first decade of life is 90% or higher [1,2], implying that most primary infections occur during childhood. In immunocompromised patients, reactivation of a latent infection is frequently observed [1,3] as BK virus nephropathy (BKVN) or hemorrhagic cystitis [1,4]. While hemorrhagic cystitis frequently develops in patients with hematologic stem cell transplantation, BKVN is essentially a complication of kidney transplantation [2,5]. BKVN has recently gained clinical significance with the introduction of potent immunosuppressive agents. The prevalence of BKVN in adult kidney allograft recipients is 1% to 10% [2,5,6] and has been reported to be 2% to 8% in pediatric renal transplant recipients [7,8]. BKVN is considered to be an early complication of kidney transplantation that often occurs in the first year of transplantation; 95% of BKVN develops within two years of transplantation according to the organization Kidney Disease: Improving Global Outcomes (KDIGO), but it may develop as late as in the fifth year [9,10,11]. Importantly, after BKVN is diagnosed, over 15% of patients are expected to lose their allograft kidney within one year [9].

Risk factors for BKVN in kidney allograft recipients include older age, male sex, ethnicity (non-African American), increased number of human leukocyte antigen (HLA) mismatches, prolonged cold-ischemia time, ureteral stent placement, immunosuppression induction with anti-thymocyte globulin, tacrolimus- and/or mycophenolate mofetil-based maintenance immunosuppression, and prior rejection history [3,12]. The most important risk factor for BKVN is considered to be the degree of immunosuppression [12]. However, risk factors for BKVN in children have not yet been studied sufficiently. In a retrospective cohort study of children, a seronegative status for BKV in recipients was associated with BKVN [13].

To gain a better understanding of BKVN in pediatric kidney transplantation recipients, the clinical characteristics and risk factors for BK virus infection and BKVN in pediatric kidney allograft recipients was assessed in this study.

## 2. Methods

### 2.1. Study Population and Ethics

We retrospectively reviewed the medical records of all pediatric kidney allograft recipients who underwent transplantation at the Seoul National University Hospital between January 2001 and July 2015. Clinical findings until July 2018 were assessed. This study was approved by the Institutional Review Board of the Seoul National University Hospital (IRB no. 1808-156-967) and was conducted in accordance with the Declaration of Helsinki.

### 2.2. Immunosuppression

The immunosuppression protocol of the Seoul National University Hospital consisted of steroids, tacrolimus, and mycophenolate mofetil. Methylprednisolone was administered as a 10 mg/kg intravenous bolus dose at the time of surgery and was tapered gradually to a maintenance dose of prednisolone 0.3 mg/kg by one month after transplantation. In patients with a low risk of rejection, prednisolone was discontinued by one year after transplantation. The tacrolimus target trough level was 8–12 ng/mL for up to three months, 6–8 ng/mL until six months, and 4–6 ng/mL thereafter. From 2001 to 2008, basiliximab was used as an induction therapy for high-risk patients with a transplanted kidney from a deceased donor or a high number of HLA mismatches. After 2008, all patients received basiliximab induction therapy.

### 2.3. BK Viremia and BKVN

BK virus DNA in the plasma of patients was tested by polymerase chain reaction (PCR) to detect the large T antigen of BKV (BKV ELITe kit, ELITechGroup, Puteaux, France). BK viral load quantification became available as of June 2008 and thereafter BK viral load of >100 copies/mL was considered as BK viremia. In principle, since 2008, BK viremia was screened every month for three months after transplantation, then every three months for one year, and then every year up to five years. BK virus was additionally screened in patients with an unexplained acute rise in serum creatinine or in patients receiving acute rejection treatment. Upon detection, BK viremia was followed every month. Persisting BK virus PCR loads over 10,000 copies/mL for >3 weeks was categorized as presumptive BKVN, as described previously [6]. Presumptive BKVN and pathologically proven BKVN were collectively categorized as BKVN to assess the risk factors for BKVN in this study. Histologic grading of BKVN was classified according to the criteria of the University of Maryland, USA [14].

### 2.4. Statistical Analysis

SPSS version 23.0 (SPSS, Armonk, NY, USA) was used for data analysis. Categorical variables were analyzed using the Pearson chi-square test or Fisher exact test and continuous variables were compared using the *t*-test or Mann–Whitney U test. All values were reported as a median (range). To assess risk factors for BKVN, univariate analysis was performed using the Kaplan–Meier test with the log-rank test and multivariate analysis was done using the Cox proportional hazards model. Factors with a value of *p* < 0.25 in the univariate analysis were included in the multivariate analysis. A value of *p* < 0.05 was considered statistically significant.

## 3. Results

**Figure 1:**
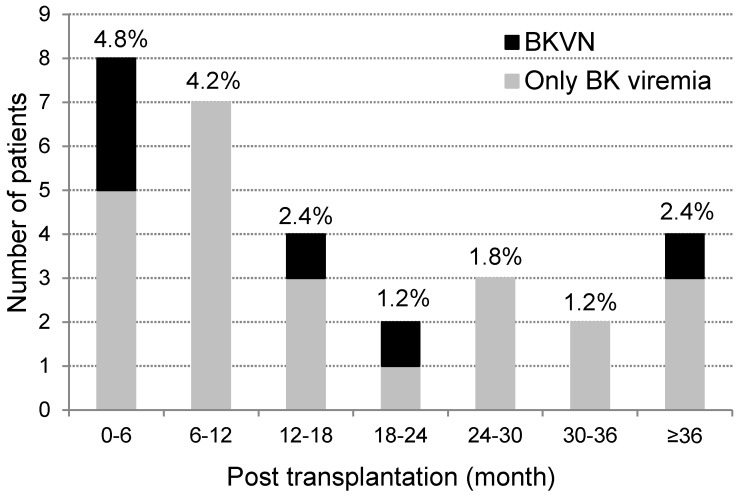
Onset of BK viremia after kidney transplantation. Values are represented as number of patients (% of total subject population).

A total of 195 patients younger than 20 years underwent allograft kidney transplantation between January 2001 and July 2015, and of these, 168 were tested for BK virus by PCR more than once (Table 1). BK viremia was positive in 30 patients (17.9%, 30/168) at 12.6 months (0.4–73.1 months) after transplantation (Figure 1). BKVN was diagnosed in six patients (3.6%, 6/168, BKVN group) at 13.4 months (3.9–60.0 months) after transplantation, with a BK viral titer at first detection of 46,508 copies/mL (16,924–289,699 copies/mL) at 10.9 months (1.5–60.0 months) and 306,277 copies/mL (41,914–10,165,852 copies/mL) at the peak at 14.2 months (4.3–60.3 months). The BK viral titer in the 24 patients with BK viremia but not BKVN (only-BK viremia group) was 437 copies/mL (119–6794 copies/mL) at first detection at 12.6 months (0.4–73.1 months) after kidney transplantation, and increased to 561 copies/mL (123–42,288 copies/mL) copies/mL at 15.3 months (0.4–73.7 months).

### 3.1. Risk Factors for BKVN and BK Viremia

To assess the risk factors for BKVN, the BKVN group (*n* = 6) and the remaining patients—including the BK viremia group and those who had not shown BK viremia (*n* = 162, non-BKVN group, Table 1)—were compared. There were no statistically significant differences in sex, age at transplant, primary kidney disease, donor source, HLA mismatch, induction with polyclonal or monoclonal antibody, prior acute rejection, and Epstein–Barr virus or cytomegalovirus infection. None of the BKVN patients had a ureteral stent placed after kidney transplantation. Interestingly, as a primary kidney disease of their native kidneys, Alport syndrome was significantly more common in the BKVN group compared to the non-BKVN group (50% vs. 4.3%, *p* = 0.003). Multivariate analysis using Cox proportional analysis also showed that Alport syndrome was a significant risk factor for BKVN (Table 2).

Comparison of the BK viremia group and non-BK viremia group revealed that basiliximab induction therapy and transplant in the years after 2008 were significantly higher in the BK viremia group than in the non-BK viremia group (Table S1). Multivariable Cox hazards regression analysis showed that induction with basiliximab was a significant risk factor for the development of BK viremia (Table S2).

Comparison between the BKVN group and the non-BKVN BK viremia group (BK viremia-only group) revealed no significant differences between the two groups, except for BK viral load and Alport syndrome (Table S3).

### 3.2. Clinical Course of BKVN

Among the six patients in the BKVN group, four had pathologically-proven BKVN and the remaining two presented with presumptive BKVN. BKVN was managed with the reduction of immunosuppressive medications, intravenous immunoglobulin, leflunomide, ciprofloxacin, or cidofovir (Table 3). Three of the six patients had a pathological diagnosis of acute rejection along with BKVN on an allograft kidney biopsy, and were also treated with intravenous methylprednisolone.

After a 2.2–8.3-year follow-up, no patients experienced graft loss, but impairment of renal function was evident (median estimated glomerular filtration rate 39.9 (range, 20.7–56.9) mL/min/1.73 m^2^). BK viremia was only cleared in three patients over a median of 20.5 months (range, 16.6–89.9 months) after the first detection of viremia.

### 3.3. Alport Syndrome and BKVN

**Figure 2:**
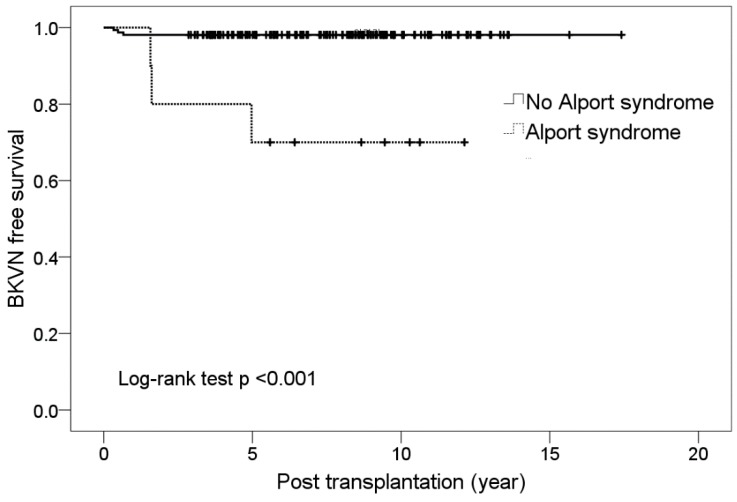
Kaplan–Meier curves indicating progression to BK virus nephropathy after renal transplantation.

Although Alport syndrome comprised 6% of the study population (10 of 168 patients), as the primary disease of the native kidneys it accounted for 50% of BKVN patients. The prevalence of BK viremia was 30% (3 of 10 patients) for Alport syndrome, and all BK viremia in patients with Alport syndrome progressed to BKVN, whereas BK viremia was found in 17.1% of the remaining patients (other than Alport syndrome as their primary disease, 27 of 158 patients), and only 11.1% of those with BK viremia progressed to BKVN. In addition, BK viremia was detected relatively late in patients with Alport syndrome at 16, 24, and 60 months after transplantation (Table 3), and the initial viral load was higher, with a median 68,919 copies/mL (versus 4773 copies/mL in others with BK viremia, *p* = 0.001). BK viremia did not resolve in these patients, despite treatment for more than 2.2 years. BKVN-free survival curves of individuals with Alport syndrome and other patient groups also showed significant differences (Kaplan–Meier analysis, log-rank test *p* < 0.001, Figure 2).

## 4. Discussion

In our study, the prevalence of BK viremia was 17.8% in pediatric kidney allograft recipients, which was similar to that shown in previous studies in adults (11% to 25%) [4,11,15] and pediatric kidney recipients (21%) [16]. The prevalence of BKVN in our pediatric kidney transplantation recipients was 3.6%, which was similar to the 4.6% reported by the North American Pediatric Renal Trials and Collaborative Studies registry [8]. BK viremia was observed immediately following kidney transplantation (0.4 months) in a minority of patients, but most cases occurred sometime after the surgery. This suggested that most patients may have contracted BKV from their peers, as primary infections of BK virus usually occur in childhood [17]. However, the pre-donation BKV status of donors had not been assessed at Seoul National University Hospital until recently, and therefore data was unavailable to help assess the source of the BK virus infection. Interestingly, all patients with BKVN received their allograft kidney after 2009 and were older than seven years; however, neither of these parameters were statistically significant, likely due to the small size of the study population. Nevertheless, regarding why BKVN only occurred after 2009, it was speculated that: (1) BK monitoring was more aggressive after 2009, as was the availability of BK viremia quantitation protocols, and (2) immunosuppression became more potent with the awareness of antibody-mediated rejection associated with insufficient immunosuppression. BKVN in individuals older than seven years may reflect the timing of primary infection or a selection bias resulting from the small pool of younger recipients.

In this study, previously known risk factors in the adult population such as older age and type of immunosuppression were not identified as risk factors for BK viremia and BKVN. This was likely due to the relatively homogenous population in the study, as the Korean pediatric population evaluated mostly underwent induction treatment with monoclonal antibodies and maintenance treatment with tacrolimus and mycophenolate mofetil. Male sex and an increased number of HLA mismatches were not significant factors in this population, although there were no BKVN patients with zero HLA mismatches. Data relative to ischemia time was not available in this study, but the distribution of donor types did not differ between the groups, implying that ischemia time was not associated with BKVN in this population. Ureteral stent placement was previously indicated as a significant risk factor for BKVN [12], but in this study, no BKVN patients were subjected to ureteral stent placement. Acute rejection was more common in the BK viremia group and in the BKVN group compared with the BK viremia-free group, but there was no statistically significant difference either. While previous studies showed that the primary cause of end-stage renal disease was not a risk factor for BKVN in children [8,13,18], in this study population, Alport syndrome was a risk factor for BKVN. Alport syndrome is caused by a genetic defect in type IV collagen which comprises basement membranes. It is a rare hereditary disorder with an incidence of 1 in 50,000 persons [19], but it is relatively common in the pediatric population as a cause of end-stage renal disease compared to the adult population. Although Alport syndrome has not been previously reported to be a risk factor for BKVN, a study of kidney transplantations in Australia and New Zealand from 1965 to 2010 reported that BKVN (*n* = 6) was found only in the group with Alport syndrome, and not in the group without Alport syndrome in a group of 243 patients [20].

Thus, the question arises: how can the finding that Alport syndrome is a risk factor of BKVN be explained? Interestingly, BKVN developed in every patient with BK viremia if the patient had Alport syndrome, while only one-tenth of patients with BK viremia progressed to BKVN (3 of 27) when Alport syndrome patients were excluded. Hence, it is speculated that tubular cells in Alport syndrome patients might be more prone to BK virus propagation, possibly because of the defective distal tubular basement membrane [21]. The gradual repopulation of recipient cells in the allograft kidney has been documented previously [22]; therefore, the vulnerability of individuals with Alport syndrome might appear gradually, which might explain the delayed occurrence of BKVN. Alternatively, BK viremia screening might not be performed sufficiently early in these patients, resulting in the propagation of BKV irrespective of immunosuppression, and later the development of BKVN. Nonetheless, further study with larger populations is necessary to validate the notion that Alport syndrome is a risk factor for BKVN.

Screening for BK viremia is recommended for the prevention and early detection of BKVN [11]. Guidelines recommend screening for BK viremia regularly (every month for three to six months after transplantation, then every three months for one or two years, and then every year up to five years) with creatinine elevation and after acute rejection treatment [10]. Considering that Alport syndrome was a significant risk factor for BKVN in this study, a more meticulous screening for Alport syndrome patients is recommended, and for a longer period after kidney transplantation [23]. When BK viremia is detected from screening, it is recommended that immunosuppression be reduced [24,25]. In this study, by reducing immunosuppression, most cases of BK viremia resolved and did not progress to BKVN. Once BKVN is diagnosed, there are a few treatment options available in addition to the reduction of immunosuppression [2,6,10], with intravenous immunoglobulin treatment, fluoroquinolone, cidofovir, and leflunomide being reported as partially effective [26]. Most of our BKVN patients were treated with these methods and no allografts were lost. Nevertheless, neither allograft renal function of BKVN patients nor eradication of BK viremia was satisfactory, as both persisted in three patients with Alport syndrome.

A shortcoming of this study is that it was a retrospective study of a small patient population. Immunosuppression protocols have changed over time, and this factor could not be controlled well. In addition, not all the cases of BKVN were confirmed by an allograft kidney biopsy. Nonetheless, considering the pediatric focus of the study, the population size was appreciable, and a relatively good outcome of BKVN was evident with the application of aggressive treatment.

## 5. Conclusions

The incidence of BKVN was 3.6% in pediatric kidney allograft recipients at Seoul National University Hospital, and BKVN was associated with the underlying disease, Alport syndrome. Following aggressive treatment, no BKVN cases resulted in the loss of an allograft kidney for over two years. Further study with a larger population is necessary to validate the notion that Alport syndrome is a risk factor for BKVN.

## Figures and Tables

**Table 1:** Baseline characteristics of patients with or without BK virus nephropathy.

Characteristic	BKVN (*n* = 6)	Non-BKVN (*n* = 162)	Total (*n* = 168)	*p*-Value
Sex, M:F	3:3	98:64	101:67	0.684
Recipient age at transplant, years	13.9 (7.7–18.9)	13.2 (1.5–19.9)	13.3 (1.5–19.9)	0.587
Primary kidney disease				
Glomerulopathy	5 (83.3)	85 (52.5)	90 (53.6)	0.282
CAKUT	0	36 (22.2)	36 (21.4)	
Other	1	41 (25.3)	42 (25.0)	
Alport syndrome	3 (50.0)	7 (4.3)	10 (6.0)	0.003
Donor type				
Deceased donor	3 (50.0)	63 (38.9)	66 (39.3)	0.681
Living donor	3 (50.0)	99 (61.1)	102 (60.7)	
Recipient age at transplant, years				
0 to <7	0	28 (17.3)	28 (16.7)	0.285
7 to <13	2 (33.3)	52 (32.1)	54 (32.1)	
≥13	4 (66.7)	82 (50.6)	86 (51.2)	
HLA mismatch				
0–2	0	35 (21.6)	35 (20.8)	0.578
3–4	6 (100)	112 (69.1)	118 (70.2)	
5–6	0	15 (9.3)	15 (8.9)	
Acute cellular rejection	4/6 (66.7)	95/151 (62.9)	99/157 (63.1)	1.000
Immunosuppression				
MMF	6	162	168	1.000
Tac	6	161 (99.4)	167 (99.4)	1.000
BSX	6	131 (80.9)	137 (81.5)	0.594
ATG	0	9 (5.6)	9 (5.4)	1.000
Transplant year				
2001–2008	0	47 (29.0)	47 (28.0)	0.187
2009–2015	6	115 (71.0)	121 (72.0)	
CMV infection	3/5 (60)	60/131 (45.8)	63/136 (46.3)	0.663
EBV infection	1/4 (25)	70/132 (53.0)	71/136 (52.2)	0.348
*Pneumocystis jirovecii* pneumonia	0	6 (3.7)	6 (3.6)	1.000
PTLD	0	8 (4.9)	8 (4.8)	1.000
Comorbidity				
Hypertension	2 (33.3)	27 (16.7)	29 (17.3)	0.277
Cardiovascular disease ^1^	0	8 (4.9)	8 (4.8)	1.000
Diabetes mellitus	0	9 (5.6)	9 (5.4)	1.000
Dyslipidemia	0	10 (6.2)	10 (6.0)	1.000
Neurological disorder ^2^	0	14 (8.6)	14 (8.3)	1.000
Liver disease ^3^	1 (16.7)	7 (4.3)	8 (4.8)	0.257
Cancer except PTLD	0	1 (0.6)	1(0.6)	1.000
Mortality	0	2 (1.2)	2 (1.2)	1.000

^1^ Cardiomyopathy, myocarditis, congenital heart defect, and myocardial infarction. ^2^ Developmental delay, congenital malformations of the nervous system, and epilepsy. ^3^ Hepatitis, fatty liver, congenital hepatic fibrosis, and liver cirrhosis. Values are expressed as numbers (%) and median (range). Abbreviations: BKVN: BK virus nephropathy; CAKUT: congenital anomalies of the kidney and the urinary tract; HLA: human leukocyte antigen; MMF: mycophenolate mofetil; Tac: tacrolimus; BSX: basiliximab; ATG: anti-thymocyte globulin; CMV: cytomegalovirus; EBV: Epstein–Barr virus; PTLD: post-transplant lymphoproliferative disease.

**Table 2:** Risk factors for BK virus nephropathy.

Factors	Univariate	Multivariate ^1^	
	*p*-Value	Hazard Ratio (95% CI)	*p*-Value
Alport syndrome	<0.001	13.204 (2.662–65.502)	0.002
HLA mismatch ≥3	0.206	NS	0.984
Basiliximab	0.224	NS	0.975
Transplant year, 2009–2015	0.108	NS	0.976

^1^ Factors with a value of *p* < 0.25 in the univariate analysis were included in the multivariate analysis. Abbreviations: CI: confidence interval; NS: not significant; HLA: human leukocyte antigen.

**Table 3:** The clinical course of patients with BKVN.

Patient No.	Sex/Age at BKVN (yr)	Primary Disease	Transplant Year/Donor Type	Onset of Viremia after KT	Initial Level ^1^ (Copies/mL)	Peak Level ^2^ (Copies/mL)	Biopsy Grade	Treatment	Follow up after BKVN	Time to Viremia Clearance
1	F/8.0	ATN	2009/D	1.5 mo	16,924	10,165,852	B1	Reduction of IS, IVIG, leflunomide	8.3 yr	89.9 mo
2	F/20.6	Alport	2010/D	60.3 mo	289,699	289,699	B3	Reduction of IS, IVIG, leflunomide	3.0 yr	No
3	M/13.1	Alport	2010/L	24.0 mo	41,914	41,914	B1	Reduction of IS, IVIG, leflunomide	6.4 yr	No
4	M/15.0	Alport	2014/L	15.9 mo	68,919	2,266,980	B1	Reduction of IS, IVIG, leflunomide, ciprofloxacin, cidofovir	2.2 yr	No
5	F/16.8	FSGS	2014/L	4.6 mo	51,102	322,854	ND	Reduction of IS, leflunomide, ciprofloxacin	3.8 yr	16.6 mo
6	M/19.5	FSGS	2015/D	6.0 mo	25,555	62,481	ND	Reduction of IS, leflunomide	2.4 yr	20.5 mo

^1^ The value of BK virus load when BK viremia was first detected. ^2^ The highest value of BK virus load. Abbreviations: BKVN: BK virus nephropathy; S-Cr: serum creatinine; ATN: acute tubular necrosis; VUR: vesicoureteral regurgitation; FSGS: focal segmental glomerulosclerosis; D: deceased; L: living; ND: not done; IS: immunosuppressant; IVIG: intravenous immunoglobulin; mo: months; yr: year.

## Supplementary Materials

The following are available online at https://www.mdpi.com/2077-0383/8/4/491/s1. Table S1: Baseline characteristics of patients with and without BK viremia, Table S2: Risk factors for BK viremia, Table S3: Comparison between the BKVN group and only-BK viremia groups.
