# Supplementary material for: Higher Incidence of BK Virus Nephropathy in Pediatric Kidney Allograft Recipients with Alport Syndrome
Source: J Clin Med. 2019 Apr 11;8(4):491. doi: 10.3390/jcm8040491 (PMC6517952; doi:10.3390/jcm8040491)
Supplement: Supplementary file 1 [file jcm-08-00491-s001.pdf]

Table 1. Baseline characteristics of patients with and without BK viremia.

Characteristic	BK Viremia (*n* = 30)	Non-BK Viremia (*n* = 138)	Total (*n* = 168)	*p*-Value
Sex, M:F	19:11	82:56	101:67	0.692
Recipient age at transplant, years	13.3 (2.0–19.9)	13.3 (1.5–19.9)	13.3 (1.5–19.9)	0.720
Primary kidney disease				
Glomerulopathy	19 (63.3)	71 (51.4)	90 (53.6)	0.111
CAKUT	8 (26.7)	28 (20.3)	36 (21.4)	
Others	3 (10.0)	39 (28.3)	42 (25.0)	
Alport syndrome	3 (10.0)	7 (5.1)	10 (6.0)	0.387
Donor type				
Deceased donor	16 (53.3)	50 (36.2)	66 (39.3)	0.082
Living donor	14 (46.7)	88 (63.8)	102 (60.7)	
Recipient age at transplant, years				
0 to <7	4 (13.3)	24 (16.9)	28 (16.7)	0.864
7 to <13	10 (33.3)	48 (33.8)	54 (32.1)	
≥13	16 (53.3)	70 (49.3)	86 (51.2)	
HLA mismatch				
0–2	6 (20.0)	29 (21.0)	35 (20.8)	0.471
3–4	23 (76.7)	95 (68.8)	118 (70.2)	
5–6	1 (3.3)	14 (10.1)	15 (8.9)	
Acute cellular rejection	20/30 (66.7)	79/127 (62.2)	99/157 (63.1)	0.649
Immunosuppression				
MMF	30	138	168	1.000
Tac	30	137 (99.3)	167 (99.4)	1.000
BSX	29 (96.7)	108 (78.3)	137 (81.5)	0.019
ATG	2 (6.7)	7 (5.1)	9 (5.4)	0.663
Transplant year				
2001–2008	4 (13.3)	43 (31.2)	47 (28.0)	0.049
2009–2015	26 (86.7)	95 (68.8)	121 (72.0)	
CMV infection	14/27 (51.9)	49/109 (45.0)	63/136 (46.3)	0.520
EBV infection	11/24 (45.8)	60/112 (53.6)	71/136 (52.2)	0.491
Pneumocystis jirovecii pneumonia	3 (10.0)	3 (2.2)	6 (3.6)	0.071
PTLD	1 (3.3)	7 (5.1)	8 (4.8)	1.000
Comorbidity				
Hypertension	5 (16.7)	24 (17.4)	29 (17.3)	1.000
Cardiovascular disease [1]	2 (6.7)	6 (4.3)	8 (4.8)	0.634
Diabetes mellitus	1(3.3)	8 (5.8)	9 (5.4)	1.000
Dyslipidemia	0	10 (7.2)	10 (6.0)	0.212
Neurological disorder [2]	3 (10.0)	11 (8.0)	14 (8.3)	0.718
Liver disease [3]	3 (10.0)	5 (3.6)	8 (4.8)	0.153
Cancer except PTLD	0	1 (0.7)	1 (0.6)	1.000
Mortality	1 (3.3)	1 (0.7)	2 (1.2)	0.326

[1]Cardiomyopathy, myocarditis, congenital heart defect, and myocardial infarction. [2]Developmental delay, congenital malformations of the nervous system, and epilepsy. [3]Hepatitis, fatty liver, congenital hepatic fibrosis, and liver cirrhosis. Values are expressed as numbers (%) and median (range). Abbreviations: BKVN, BK virus nephropathy; CAKUT, congenital anomalies of the kidney and the urinary tract; MMF, mycophenolate mofetil; Tac, tacrolimus; ATG, anti-thymocyte globulin; BSX, basiliximab; CMV, cytomegalovirus; EBV, Epstein-Barr virus; PTLD, post-transplant lymphoproliferative disease

Table S2. Risk factors for BK viremia

	**Univariate**	**Multivariate[1]**	
**Factors**	***P*-value**	**HR (95% CI)**	***P*-value**
Primary kidney disease, glomerulopathy	0.192	1.702 (0.808–3.583)	0.162
Donor type, deceased	0.095	1.561 (0.741–3.288)	0.241
Basiliximab	0.015	8.069 (1.097–59.361)	0.040
Transplant year, 2009–2015	0.029	2.149 (0.734–6.296)	0.163

[1]Factors with a value of P <0.25 in the univariate analysis were included in the multivariate analysis.
Abbreviations: HR, hazard ratio; CI, confidence interval

Table S3. Comparison between the BKVN group and only-BK viremia groups

**Characteristic**	**BKVN (*n* = 6)**	**Only-BK viremia (*n* = 24)**	**Total (*n* = 30)**	***P*-value**
Sex, M:F	3:3	16:8	19:11	0.641
Recipient age at transplant, years	14.5 (7.7–18.9)	12.7 (2.0–19.9)	13.3 (2.0–19.9)	0.667
Primary kidney disease				
Glomerulopathy	5	14 (58.3)	19 (63.3)	0.275
CAKUT	0	8 (33.3)	8 (26.7)	
Others	1	2 (8.3)	3 (10.0)	
Alport syndrome	3 (50)	0	3 (10.0)	0.005
Donor type				
Deceased donor	3 (50)	13 (54.2)	16 (53.3)	1.000
Living donor	3 (50)	11 (45.8)	14 (46.7)	
Recipient age at transplant, years				
0 to <7	0	4 (16.7)	4 (13.3)	0.830
7 to <13	2 (33.3)	8 (33.3)	10 (33.3)	
≥13	4 (66.7)	12 (50)	16 (53.3)	
HLA mismatch				
0–2	0	6 (25.0)	6 (20.0)	0.436
3–4	6	17 (70.8)	23 (76.7)	
5–6	0	1 (4.2)	1 (3.3)	
Acute cellular rejection	4/6 (66.7)	16/24 (66.7)	20/30 (66.7)	1.000
Immunosuppression				
MMF	6	24	30	1.000
Tac	6	24	30	1.000
BSX	6	23 (95.8)	29 (96.7)	1.000
ATG	0	2 (8.3)	2 (6.7)	1.000
Transplant year				
2001–2008	0	5 (20.8)	5 (16.7)	0.557
2009–2015	6	19 (79.2)	25 (83.3)	
CMV infection	3/5 (60.0)	11/22 (50)	14/27 (51.9)	1.000

EBV infection	1/4 (25.0)	10/20 (50)	11/24 (45.8)	0.596
Pneumocystis jirovecii pneumonia	0	3 (12.5)	3 (10.0)	1.000
PTLD	0	1 (4.2)	1 (3.3)	1.000
Comorbidity				
Hypertension	2 (33.3)	3 (12.5)	5 (16.7)	0.254
Cardiovascular disease[1]	0	2 (8.3)	2 (6.7)	1.000
Diabetes mellitus	0	1 (4.2)	1 (3.3)	1.000
Dyslipidemia	0	0	0	1.000
Neurological disorder[2]	0	3 (12.5)	3 (10.0)	1.000
Liver disease[3]	1 (16.7)	2 (8.3)	3 (10.0)	0.501
Cancer except PTLD	0	0	0	1.000
Mortality	0	1 (4.2)	1 (3.3)	1.000
Initial BKV level, copies/mL	46,508 (16,924–289,699)	437 (119–6,794)	699 (119–289,699)	<0.001
Peak BKV level, copies/mL	306,277 (41,914–10,165,825)	561 (123–42,288)	905 (123–10,165,825)	<0.001
Tac dose at BK viremia, mg/kg/day	0.135 (0.053–0.330)	0.128 (0–0.369)	0.127 (0–0.369)	0.432
Laboratory findings at BK viremia				
Tac level, ng/mL	6.0 (3.3–7.4)	7.1 (2.6–14.2)	6.3 (2.6–14.2)	0.230
Estimated GFR, mL/min/1.73m^2	43.2 (37.6-60.1)	67.5 (31.1-103.3)	63.4 (31.1-103.3)	0.065
Hematuria	1 (16.7)	5 (20.8)	6 (20.0)	1.000
Proteinuria	0	3 (12.5)	3 (10.0)	1.000

[1]Cardiomyopathy, myocarditis, congenital heart defect, and myocardial infarction. [2]Developmental delay, congenital malformations of the nervous system, and epilepsy. [3]Hepatitis, fatty liver, congenital hepatic fibrosis, and liver cirrhosis. Values are expressed as numbers (%) and median (range). Abbreviations: BKVN, BK virus nephropathy; CAKUT, congenital anomalies of the kidney and the urinary tract; MMF, mycophenolate mofetil; Tac, tacrolimus; ATG, anti-thymocyte globulin; CMV, cytomegalovirus; EBV, Epstein-Barr virus; BKV, BK virus; PTLD, post-transplant lymphoproliferative disease GFR, glomerular filtration rate
